# Anabolic Hormone Deficiencies in Heart Failure with Reduced or Preserved Ejection Fraction and Correlation with Plasma Total Antioxidant Capacity

**DOI:** 10.1155/2020/5798146

**Published:** 2020-01-03

**Authors:** Antonio Mancini, Angela Maria Rita Fuvuzzi, Carmine Bruno, Maria Anna Nicolazzi, Edoardo Vergani, Nunzia Ciferri, Andrea Silvestrini, Elisabetta Meucci, Nicola Nicolotti, Roberta D'Assante, Antonio Cittadini

**Affiliations:** ^1^Operative Unit of Endocrinology, Fondazione Policlinico Universitario A Gemelli IRCCS, Università Cattolica del Sacro Cuore, Rome, Italy; ^2^Operative Unit of Internal Medicine and Vascular Diseases, Division of Internal Medicine and Cardiovascular Diseases, Fondazione Policlinico Universitario A Gemelli IRCCS, Università Cattolica del Sacro Cuore, Rome, Italy; ^3^Istituto di Biochimica e Biochimica Clinica, Università Cattolica del Sacro Cuore, Rome, Italy; ^4^Medical Management, Fondazione Policlinico Universitario A. Gemelli IRCCS, Rome, Italy; ^5^Department of Translational Medical Sciences, Federico II University of Naples, Naples, Italy

## Abstract

**Background:**

While anabolic hormone deficit is a common finding in heart failure with reduced ejection fraction (HFrEF), few data are available in heart failure with preserved ejection fraction (HFpEF).

**Methods:**

Blood samples were collected for metabolic (total cholesterol, HDL cholesterol, LDL cholesterol, creatinine, and glucose) and hormonal (IGF-1, DHEA-S, TSH, fT3, fT4, and T) determination, comparing 30 patients with HFpEF and 20 patients with HFrEF. Total antioxidant capacity was evaluated by using the spectrophotometric method using the latency time in the appearance of the radical species of a chromogen (LAG, sec) as a parameter proportional to antioxidant content of the sample. Echocardiographic parameters were also assessed in the two groups.

**Results:**

A high prevalence of testosterone (32% in HFrEF and 72% in HFpEF, *p* < 0.05) and DHEA-S deficiencies was observed in HFpEF patients. Echocardiographic parameters did not correlate with hormone values. A significant direct correlation between T (*r*^2^ = 0.25, *p* < 0.05) and DHEA-S (*r*^2^ = 0.19, *p* < 0.05) with LAG was observed only in HFpEF.

**Conclusion:**

Anabolic hormone deficiency is clearly shown in HFpEF, as already known in HFrEF. Although longitudinal studies are required to confirm the prognostic value of this observation, our data suggest different mechanisms in modulating antioxidants in the two conditions, with possible therapeutic implications.

## 1. Introduction

Chronic heart failure (CHF) is defined as a clinical syndrome based on an unbalance between cardiac output and metabolic requirements of organism [1]. Any structural or functional disorders such as coronary heart disease, hypertension, diabetes, cardiomyopathies, heart valve diseases, arrhythmias, congenital heart defects, anaemia, cocaine abuse, AIDS, thyroid disorders, radiation, and chemotherapy that reduce the ability of the ventricle to fill with or eject an adequate volume of blood may be the cause of this condition. It is a staggering plague for our times since its prevalence is around 1-2% of the adult population in developed countries, with a peak ≥10% among people >70 years of age [2–4].

The main classification of CHF relies on left ventricular ejection fraction (LVEF), evaluated by echocardiography, or, less frequently, myocardial scintigraphy and magnetic resonance of the heart; its measurement identifies three classes of CHF: from the well known and classic heart failure with reduced ejection fraction (HFrEF), which includes patients with left ventricular ejection fraction (LVEF) <40%, to heart failure with preserved ejection fraction (HFpEF), which comprises patients with LVEF <50%, to the grey area of LVEF in the range of 40–49%, which describes the new entity of heart failure with midrange ejection fraction [1]. HFrEF and HFpEF are the most known subtypes, with a relevant and increasing literature concerning the last one [5, 6]. They are different syndromes, with different pathogenesis, pathophysiologies, and therapy. In HFrEF, the hinge point is a direct damage to the heart that leads to reduced left ventricle contraction [7], whereas in HFpEF, diastolic dysfunction is the main mechanism involved, with other features contributing to this scenario such as left atrial dysfunction, right ventricular dysfunction, pulmonary hypertension, and increased vascular stiffness [8–12].

Both the conditions present with a high prevalence of multihormonal deficiencies [13, 14]. The impairment of major anabolic systems (somatotropic, adrenal, and gonadal) does not appear to represent a mere epiphenomenon but is involved in the CHF pathophysiology; especially low serum testosterone (T), dehydroepiandrosterone-sulfate (DHEA-S), and insulin-like growth factor (IGF)-1 levels have been correlated to the symptoms severity and the adverse outcomes in men suffering from CHF [15–19]. On the contrary, T, DHEA-S, and IGF-1 are known to regulate oxidative stress in different manners [20], exerting pro-oxidative effects or exhibiting an antioxidant power. CHF is a syndrome in which inflammation and OS play a fundamental role, and, in turn, this may point to a pivotal role of these hormonal alterations both in the pathogenesis of CHF and in its treatment.

Total antioxidant capacity (TAC) expresses the whole effects of nonproteic nonenzymatic antioxidants, as widely discussed in previous studies [21, 22]. Previously, we have shown the modulatory action of anabolic hormones on this parameter and its variations in CHF [20, 23].

Thus, the aim of the present study was to explore the correlation between anabolic hormones, echocardiographic parameters, and TAC in HFpEF and HFrEF and correlate them with metabolic parameters (with the aim to better understand the possible molecular consequences of hormonal derangement in these conditions).

## 2. Materials and Methods

50 subjects involved in this study were admitted to the University Hospital “Policlinico Gemelli” Dept. of Internal Medicine and were enrolled after being given an explanation of purposes and nature of the study, conducted in accordance with the Declaration of Helsinki, as revised in 2013. The study protocol was approved by the Institutional review board of “Medical Pathology” of our University Hospital.

Twenty patients with HFrEF, aged 42–88 years (mean 69.5), and thirty patients with HFpEF, aged 59–90 years (mean 77.7), were recruited. The diagnosis of HFpEF was established according to the current guidelines of the European Society of Cardiology [1]. Patients with end-stage renal disease, liver cirrhosis, and neoplastic or autoimmune diseases were excluded. All patients were nonsmokers or had stopped smoking for at least a year. Clinical, anthropometric, and echocardiographic evaluations were achieved, including the main risk factors for cardiovascular disease. Prevalence of comorbidities (T2DM, hypertension, atrial fibrillation, peripheral atherosclerosis, non-end-stage chronic kidney disease, and COPD) was evaluated. The two groups were not significantly different for age, BMI, and NYHA classes (all belonged to class II-III).

Between 08.30 and 09.00 a.m., after an overnight fast, a polyethylene catheter was inserted into the antecubital vein of one forearm and the blood was collected using a 6 mL vacutainer blood collection tube containing lithium heparin and immediately centrifuged (4°C at 3000 ×g for 15 min) with aliquots stored at −80°C until assayed.

We evaluated metabolic (glycaemia, insulinemia, and total-HDL-LDL cholesterol) and hormonal pattern (fT3, fT4, TSH, IGF-1, T, DHEA-S, and NT-proBNP).

Fasting glucose and insulin levels were quantified with commercial kits using ADVIA automatic analyser (Siemens, Italy).

Plasmatic concentrations of NT-proBNP, TSH, fT3, fT4, DHEA-S, T, and IGF-1 were measured by using immunochemiluminometric assays on a Roche Modular E170 analyser (Roche Diagnostics, Indianapolis, IN, USA). The intra- and interassay CV for all hormones were, respectively, <5.0% and <7.0%.

Normal ranges in our laboratory were NT-proBNP (>126 pg/ml), TSH (0.4–3.2 *μ*UI/ml), fT3 (2.4–4.2 pg/ml), fT4 (8.5–16.5 pg/ml), DHEA-S (800–3500 ng/ml), and T (2.5–8.4 ng/ml). Values equal or below the lower limit of normal ranges were used to define as deficiency. For IGF-1, due to the age-related variations, we applied, to define IGF-1 deficiency, criteria of the TOSCA registry referring to the 33th percentile of a population of men with chronic heart failure (i.e., 122 ng/ml for age range under 55 years, 109 ng/ml for age range 55–64 years, 102 ng/ml for age range 65–74, and 99 ng/ml for age range older than 75 years) [13, 17].

A complete echocardiographic evaluation was performed (Echocardiography Philips, Affiniti 70c), measuring the following parameters: left ventricular ejection fraction (EF), left ventricular end-diastolic diameter (LVEDD), left ventricular end-systolic diameter (LV-ESD), left ventricular end-diastolic volume (LVEDV), left ventricular end-systolic volume (LV-ESV), septal thickness (IVS), posterior wall thickness (LV-PW), peak E-wave velocity (E), peak A-wave velocity (A), E/A ratio, pulsed-wave TDI E′ velocity (E′), E/E′ ratio, deceleration time (DT), left atrial volume (LAV), indexed left atrial volume (LAVI), systolic pulmonary artery pressure (SPAP), tricuspid annular plane systolic excursion (TAPSE), and tricuspid peak velocity (TPV).

Total antioxidant capacity (TAC) was evaluated with the method of Rice-Evans and Miller [21], modified as previously reported [24]. The method is based on the interaction between the system H_2_O_2_-metmyoglobin with the chromogen ABTS, whose radical form is spectroscopically detectable. The latency time (LAG in sec.) before the appearance of radical species is proportional to the antioxidant concentration in the sample.

HOMA-IR was used as an index of insulin resistance and was obtained from the fasting blood insulin (immunoreactive insulin (IRI)) concentration and the fasting blood sugar (FBS) level early in the morning, based on the following equation: HOMA-IR = (IRI × FBS)/405.

To estimate the sample sizes, the estimated decrease prevalence of T deficiency between the two groups was set at 20%, based on the only reported work that, at the best of our knowledge, evaluated these data in HFrEF vs HFpEF patients [14], with a type I error rate of 0.05 and a type II error rate of 0.20 (i.e., power of 0.80). Due to the expected effect size, a total of 44 patients were considered adequate.

The Mann–Whitney *U* test was employed to evaluate differences between the two groups of subjects. A *p* value of 0.05 was considered statistically significant. Linear regression analysis was employed to correlate TAC with hormonal parameters. The *X* square test was used to compare percent differences between the two groups, when considering the prevalence of hormone deficiencies and comorbidities.

## 3. Results

Table 1 shows the echocardiographic parameters: other than ejection fraction, which was different, by definition, other differences were found in LVEDV, LVESV, and LAV, all significantly higher in HFrEF (*p* < 0.05). On the contrary, the A wave was significantly higher in HFpEF (*p* < 0.05).

Comorbidities, as expected, were more prevalent in HFpEF patients (41% T2DM, 72% hypertension, 36% atrial fibrillation, 68% peripheral atherosclerosis, 63% non-end-stage chronic kidney disease, and 36% COPD) than in HFrEF patients (30% T2DM, 39% hypertension, 44% atrial fibrillation, 5% peripheral atherosclerosis, 33% non-end-stage chronic kidney disease, and 16% COPD). *X* square analysis showed a significant difference only in hypertension and peripheral atherosclerotic disease (*p* < 0.05).

Table 2 shows the metabolic and hormonal parameters in the two groups. Significant differences were observed in NT-proBNP, total cholesterol, and T, with higher levels in HFrEF. LAG values were not significantly different between the two groups despite higher prevalence of comorbidities in HFpEF.

**Figure 1:**
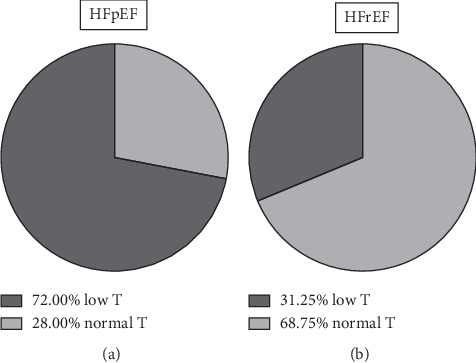
Percentage of patients with low testosterone levels in the two forms of heart failure (preserved or reduced ejection fraction, HFpEF and HFrEF, respectively).

Figure 1 shows the graphical representation of percent prevalence of T deficiency, and DHEA-S levels were under the normal range in all but two patients. The prevalence of T deficiency was significantly different between the two groups using the *X* square test (*p* < 0.05).

**Figure 2:**
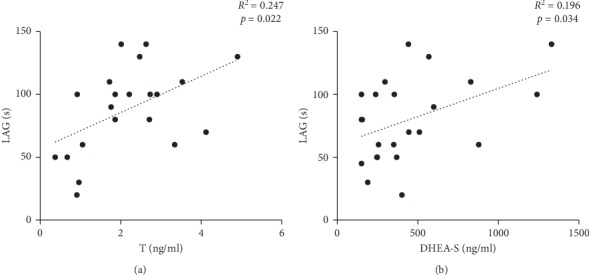
Graphical representation of correlation between LAG (latency time in the appearance of radical species, see text for the method), as a parameter of total antioxidant capacity and levels of testosterone T (a) or DHEA-S (b) in patients with heart failure with preserved ejection fraction (HFpEF).

No significant correlation was present when correlating T or DHEA-S with echocardiographic parameters. On the contrary, both T and DHEA-S significantly correlated with LAG values, but only in patients with HFpEF (Figure 2).

## 4. Discussion

Our data confirm a high prevalence of anabolic hormones deficit in the two HF subgroups, thus expanding the concept that anabolic deficiencies are a common finding not only in HFrEF but also in HFpEF, a poorly explored condition in this concern. We found a significant difference in the prevalence of low T in HFpEF; this datum is not fully in agreement with the only report of hormone evaluation in HFpEF [13]; however, the difference in age of patients could contribute to these results. Nevertheless, ageing seems not to be the only factor influencing hormone picture in these patients since T levels correlated with LAG in this specific group, suggesting a possible link between T, oxidative stress, and cardiac function.

Focusing on T levels, there are several evidences that, in HFrEF, low levels can represent a bad prognostic sign [18, 25]. At the state of knowledge, the same conclusion in HFpEF cannot be sustained due to the lack of large longitudinal studies.

We have neither found correlations between T or DHEA-S levels and echocardiographic parameters nor differences comparing patients with low or normal T and low or normal DHEA-S.

On the contrary, interesting data emerge when correlating hormonal levels with TAC. Previously, we have shown that antioxidant systems can counteract OS in patients with HFrEF when one single hormonal deficit is detected, while such compensation is less effective when more deficits ensue [20].

This is the first report on TAC in HFpEF. We have found a significant correlation between T and DHEA-S with LAG in such model, while this is not evident in HFrEF.

Two consequences can be argued from these data: first, an important role of anabolic hormones in modulating antioxidant systems in HFpEF; second, different pathophysiological mechanisms underlying the two models of HF.

For both hormones, conflicting data are reported in literature showing pro-oxidant or antioxidant effects, depending on concentration or different models studied. For instance, DHEA-S administration can induce oxidative stress in hearts of male wistar rats, defining various histologic cardiac lesions in rats, such as misshapen cell nuclei, leukocytic infiltrates, disorganized myocardial fibers, and echocardiographic alterations (increased LV-PW and LV-ESD) [26, 27], while it exerts a protective role in the liver of diabetic rats [28] and rats with obstructive jaundice [29]; in ovariectomized rats, it improves nitric oxide (NO) production, vascular function, and blood pressure levels [30]. The results of *in vivo* and *in vitro* studies have shown that DHEA-S limits lipid peroxidation [31, 32]. Moreover, oxidative stress parameters in plasma and in peripheral blood mononuclear cells in diabetic subjects are significantly decreased by DHEA-S treatment [33].

The same double-faced effects are attributed to T. In fact, ROS production in vascular smooth muscle cells in culture is stimulated by T, especially in hypertensive animal models [34]; T also stimulates xanthine oxidase and therefore superoxide generation [35]; finally, acute administration of T in supraphysiological doses increased NO urinary metabolites in healthy subjects [36]. However, it well established a protective role in ischaemic cardiopathy [37–39]; it is also known that T has a vasodilatory property via nongenomic mechanisms [40].

The literature concerning the T evaluation in CHF has been extensively reviewed, also for the therapeutic implication. T deficiency has a key role in some pathophysiological aspects of CHF, such as reduced muscle mass, abnormal energy handling, dyspnoea, and fatigue [41]. The so-called “muscle hypothesis” is based on functional and structural alterations of myocytes, which are strongly influenced by anabolic hormones [42, 43]. Metabolic influence of T has been also reported [25, 44]. However, some data are still contrasting: total and free T levels have been shown to decrease in elderly patients and related to CHF severity, but they were not independent predictors for mortality [45]. Long-term epidemiological trials were in favour of a protective effect of T treatment in the reduction of major adverse cardiovascular events and mortality [44], even if other meta-analyses raised doubts on this topic [46–49].

Our study clearly shows the correlation of the two hormones with antioxidant systems and is therefore in favour of a positive role on OS, which is one of the main players in the pathophysiology of HF.

According to literature, our patients with HFpEF, with a higher prevalence of obesity and other metabolic comorbidities, exhibited a trend toward increased LAG values. It can be speculated that a further increase in oxidative stress condition could induce a compensatory increase in antioxidant systems, possibly influencing T levels with a reciprocal vicious circle due to the modulatory role of T itself on antioxidants. Such correlation is not evident in the HFrEF group in which a worse cardiac performance or systemic catabolic status is present in CHF. Therefore, two different pathophysiological models seem to be involved in the two kinds of CHF. As recently proposed, in HFrEF, the process starts with primary ischaemic or oxidative damage of cardiomyocytes, whereas in HFpEF, a cascade of events is increased by the systemic proinflammatory state related to multiple comorbidities. The resultant endothelial damage leads to microvascular coronary inflammation and, ultimately, to myocardial dysfunction [50].

Nevertheless, there are some potential limitations of the present study. Firstly, the number of subjects is relatively small, and our findings need to be validated in a larger cohort. It is not possible to express a cause-effect relation between anabolic hormones and antioxidant status. Moreover, only one parameter of antioxidant status has been evaluated although, in our previous study, the positive effect of T replacement therapy on TAC in hypogonadal patients was described [51].

In conclusion, deficit of anabolic hormones is clearly revealed in HFpEF, as already known in HFrEF. Although longitudinal studies are needed to confirm a prognostic value of this observation, our data suggest a different mechanism in modulating antioxidant systems in these two conditions. Moreover, a possible therapeutic role of antioxidants needs to be investigated, particularly when T therapy is contraindicated.

## Figures and Tables

**Table 1:** Echocardiographic parameters in the two subgroups of heart failure, with preserved or reduced ejection fraction (HFpEF and HFrEF, respectively).

	HFpEF	HFrEF	*p*
LV-EDV (ml)	48.6 ± 1	146 ± 13	<0.05
LV-ESV (ml)	31.1 ± 1.8	91 ± 10	<0.05
LV-PW (mm)	10.9 ± 0.6	10.6 ± 0.6	n.s.
E (mm/s)	575.3 ± 74.6	347 ± 152	n.s.
A (mm/s)	789.6 ± 63.3	334 ± 110.74	<0.05
EF (%)	55.7 ± 0.8	36.91 ± 2	<0.05
E/A	0.8 ± 0.1	1.10 ± 0.38	n.s.
E/e′	11.9 ± 1.2	12.42 ± 2.86	n.s.
LAV (ml)	86.4 ± 8.9	117.86 ± 12	<0.05
TAPSE (mm)	21.3 ± 0.9	19.5 ± 1.21	n.s.
SPAP (mmHg)	35.9 ± 2.4	42 ± 4.84	n.s.

LV-EDV = left ventricular end-diastolic volume; LV-ESV = left ventricular end-systolic volume; LV-PW = left ventricular posterior wall thickness; E = E wave; A = A wave; e′ = e′ wave; EF = ejection fraction; LAV = left atrial volume; TAPSE = tricuspid annular plane systolic excursion; SPAP = systolic pulmonary arterial pressure.

**Table 2:** Mean ± SEM values of clinical, metabolic, and hormonal values in the two subgroups of heart failure, with preserved or reduced ejection fraction (HFpEF and HFrEF, respectively).

	HFpEF	HFrEF	*p*
NHYA class	II (*n* = 12)	II (*n* = 10)	
	III (*n* = 18)	III (*n* = 10)	
NT-proBNP (pg/ml)	2862.2 ± 488.5	7500.16 ± 2459.5	<0.05
Total cholesterol (mg/dl)	125.5 ± 6.9	153.6 ± 10.9	<0.05
HDL-C (mg/dl)	34.4 ± 2	44.47 ± 5.2	n.s.
LDL-C (mg/dl)	75.7 ± 7.3	91.36 ± 7.3	n.s.
Creatinine (mg/dl)	1.3 ± 0.1	1.27 ± 0.1	n.s.
Insulin (*μ*UI/ml)	11.8 ± 1.4	25.32 ± 9.9	n.s.
Glucose (mg/dl)	88.5 ± 4.7	94.95 ± 8.4	n.s.
HOMA-IR	2.8 ± 0.5	3.68 ± 1.3	n.s.
BMI (kg/m^2^)	28.7 ± 1.1	28.03 ± 1.2	n.s.
IGF-1 (ng/ml)	89.4 ± 7.1	112.78 ± 11.8	n.s.
DHEA-S (ng/ml)	438.9 ± 61.1	564.1 ± 140.3	n.s.
TSH (*μ*U/ml)	2.5 ± 0.5	2.82 ± 1.0	n.s.
fT3 (pg/ml)	2.4 ± 0.1	2.5 ± 0.1	n.s.
fT4 (pg/ml)	11.3 ± 0.5	11.28 ± 0.5	n.s.
T (ng/ml)	2.4 ± 0.2	3.55 ± 0.5	<0.05
LAG (sec)	83 ± 6.7	75.77 ± 7.5	n.s.

## Data Availability

The data used to support the findings of this study are available from the corresponding author upon request.
