# Corrigendum to “Anabolic Hormone Deficiencies in Heart Failure with Reduced or Preserved Ejection Fraction and Correlation with Plasma Total Antioxidant Capacity”

**DOI:** 10.1155/2020/5278040

**Published:** 2020-10-14

**Authors:** Antonio Mancini, Angela Maria Rita Favuzzi, Carmine Bruno, Maria Anna Nicolazzi, Edoardo Vergani, Nunzia Ciferri, Andrea Silvestrini, Elisabetta Meucci, Nicola Nicolotti, Roberta D'Assante, Antonio Cittadini

**Affiliations:** ^1^Operative Unit of Endocrinology, Fondazione Policlinico Universitario A Gemelli IRCCS, Università Cattolica del Sacro Cuore, Rome, Italy; ^2^Operative Unit of Internal Medicine and Vascular Diseases, Division of Internal Medicine and Cardiovascular Diseases, Fondazione Policlinico Universitario A Gemelli IRCCS, Università Cattolica del Sacro Cuore, Rome, Italy; ^3^Istituto di Biochimica e Biochimica Clinica, Università Cattolica del Sacro Cuore, Rome, Italy; ^4^Medical Management, Fondazione Policlinico Universitario A. Gemelli IRCCS, Rome, Italy; ^5^Department of Translational Medical Sciences, Federico II University of Naples, Naples, Italy

In the article titled “Anabolic Hormone Deficiencies in Heart Failure with Reduced or Preserved Ejection Fraction and Correlation with Plasma Total Antioxidant Capacity” [1], the name of the author Angela Maria Rita Favuzzi was given incorrectly as Angela Maria Rita Fuvuzzi. The revised authors' list is shown above.
